# CaringGuidance™ after breast cancer diagnosis eHealth psychoeducational intervention to reduce early post-diagnosis distress

**DOI:** 10.1007/s00520-019-05028-0

**Published:** 2019-08-14

**Authors:** Robin M. Lally, Kevin A. Kupzyk, Gina Bellavia, Jennifer Hydeman, Steven Gallo, Vicki S. Helgeson, Deborah Erwin, Adam C. Mills, Jean K. Brown

**Affiliations:** 1 University at Buffalo School of Nursing, Buffalo, NY USA; 2 Present Address: University of Nebraska Medical Center College of Nursing and Fred & Pamela Buffett Cancer Center, 985330 Nebraska Medical Center, Omaha, NE USA; 3 University of Nebraska Medical Center College of Nursing Biostatistician, 985330 Nebraska Medical Center, Omaha, NE USA; 4 Graduate School of Education, University at Buffalo, Buffalo, NY USA; 5 Department of Psychology, Roswell Park Cancer Institute, Buffalo, NY USA; 6 New York State Center of Excellence in Bioinformatics & Life Sciences, University at Buffalo Center for Computational Research, Buffalo, NY USA; 7 Department of Psychology, Carnegie Mellon University, Pittsburgh, PA USA; 8 Department of Oncology Health Disparity Research, Roswell Park Cancer Institute, Buffalo, NY USA; 9 Nebraska Medicine, Department of Psychology, University of Nebraska Medical Center, Omaha, NE USA; 10 School of Nursing, University at Buffalo, Buffalo, NY USA

**Keywords:** Breast cancer, Internet, Self-management, Distress, Depressive symptoms, Psychoeducation

## Abstract

**Purpose:**

Significant cancer-related distress affects 30–60% of women diagnosed with breast cancer. Fewer than 30% of distressed patients receive psychosocial care. Unaddressed distress is associated with poor treatment adherence, reduced quality of life, and increased healthcare costs. This study aimed to evaluate the preliminary efficacy of a new web-based, psychoeducational distress self-management program, CaringGuidance™ After Breast Cancer Diagnosis, on newly diagnosed women’s reported distress.

**Methods:**

One-hundred women, in five states, diagnosed with breast cancer within the prior 3 months, were randomized to 12 weeks of independent use of CaringGuidance™ plus usual care or usual care alone. The primary multidimensional outcome, distress, was measured with the Distress Thermometer (DT), the Center for Epidemiologic Studies Depression Scale (CES-D), and the Impact of Events Scale (IES) at baseline and months 1, 2, and 3. Intervention usage was continually monitored by the data analytic system imbedded within CaringGuidance™.

**Results:**

Although multilevel models showed no significant overall effects, post hoc analysis showed significant group differences in slopes occurring between study months 2 and 3 on distress (*F*(1,70) = 4.91, *p* = .03, *η*^2^ = .065) measured by the DT, and depressive symptoms (*F*(1, 76) = 4.25, *p* = .043, *η*^2^ = .053) favoring the intervention.

**Conclusions:**

Results provide preliminary support for the potential efficacy of CaringGuidance™ plus usual care over usual care alone on distress in women newly diagnosed with breast cancer. This analysis supports and informs future study of this self-management program aimed at filling gaps in clinical distress management.

## Introduction

Three and one-half million US women live with a history of breast cancer [1]. Approximately 30–60% of these women experience significant cancer-related distress [2, 3]. Multidimensional cancer-related distress manifests along a continuum from normal fears to significant anxiety, depressive symptoms, and/or depression at clinical or subclinical levels [4–6]. Approximately 50% of women experience depression [5], depressive symptoms [6], and/or anxiety [4, 5] in the acute post-diagnosis period or within the first year [5]. While depression lessens over time, the rate of depression for breast cancer survivors (BCS) remains over twice that of the general population even 5 years later [7].

The 2019 National Comprehensive Cancer Network (NCCN) Guideline for Distress Management endorses early assessment and treatment of cancer-related distress to improve treatment adherence, reduce visits and admissions, and to improve patients’ psychological wellbeing [8]. Longitudinal studies in breast cancer support the NCCN’s recommendations [9, 10]. However, institutional capacity, access to psychological care, and patient acceptance pose barriers to distress management for 70–80% of distressed patients [11–13].

### CaringGuidance™ program

*CaringGuidance™ After Breast Cancer Diagnosis* is a new unguided, web-based, psychoeducational program developed to address the need for early and accessible self-management of cancer-related distress in newly diagnosed women to overcome institutional and patient barriers [14]. CaringGuidance™ (*version 1*) www.caringguidance.org described elsewhere [15, 16] contains five modules (17 subtopics) of supportive oncology-based psychoeducation and cognitive-behavioral techniques (e.g., cognitive reframing and rehearsal, relaxation), coping skills, problem solving, communication strategies, and validation. Content is user-guided, and offers self-tailored flexibility to explore written text; 72 survivor video vignettes featuring six BCS age 30–70 years, White and Black race, with stage 0–III breast cancer; 20 thought-challenging and reflective journaling exercises; mindfulness meditation guidance; glossary; links to cancer-related resources; and discussion board.

CaringGuidance™ was designed by a multidisciplinary team of psychology and oncology professionals as well as BCS [14] to provide a place for women to mentally process automatic thoughts and emotions associated with a new breast cancer diagnosis. Content was informed by prior qualitative research interviews with newly diagnosed women [17, 18]. For example, the module *What Does This Diagnosis Mean?* is comprised of headings and associated content from the varying thoughts shared by women within days of their diagnosis such as, “I can’t stop thinking about cancer,” “I purposely try to never think about cancer,” and “Ignoring thoughts of cancer helps me feel in control.” Each heading is followed by evidence-based guidance provided in a neutral, accepting tone. Body image, receiving and accepting support, disclosure, understanding the complexity of meaning in a cancer diagnosis, managing socially constraining behaviors, and moving forward are examples of additional topics explored in the program [15, 16]. Program content grounded in data provided by newly diagnosed women during our earlier qualitative work [17, 18] is intended to support new users’ ability to explore their thoughts and feelings, compare and contrast with what other women shared, and thus receive validation.

The efficacy of Internet delivery of cognitive-behavioral techniques (CBT) is supported [19], as is CBT to reduce depression and stress in women with breast cancer [20]. Unguided Internet CBT psychosocial interventions also show promise [21–23]. To the best of our knowledge, CaringGuidance™ is one of three fully unguided, Internet psychoeducational interventions with content specific only to women with breast cancer. CaringGuidance™ is unique, however, in that it was specifically designed for the critical earliest post-diagnosis adjustment period consistent with the NCCN Guideline recommendation for early distress intervention [8], while the other interventions were designed for women months [24] to years’ post-treatment [25].

Following development and focus group testing of CaringGuidance™ [14], our team conducted this first randomized trial of the intervention. Favorable results regarding feasibility, acceptance, and satisfaction with CaringGuidance™ by newly diagnosed women were reported earlier [16]. Women with program access also reported fewer perceived social constraints than women in the control group [15].

This is a report of findings regarding the preliminary efficacy of CaringGuidance™ on the primary outcome of distress from this first randomized trial of CaringGuidance™. The hypothesis was that women newly diagnosed with breast cancer who accessed CaringGuidance™ over 12 weeks in addition to usual care would report lower levels of distress than women who had access to usual care alone. Consistent with the goal of informing a future effectiveness/implementation trial, potential modifiers of the intervention effects were also explored.

## Method

### Subjects

Study methods are described elsewhere [15, 16] and summarized here. This trial, led by a single center in Western New York, recruited subjects through distribution of Institutional Review Board–approved (#00003128) flyers in 13 cancer, radiology, and internal medicine clinics in four states within the Eastern and Midwestern United States. Advertisements were run on radio, television, newspapers, and Facebook. Community breast cancer organizations (e.g., American Cancer Society) also distributed flyers.

Eligible women were English-speaking, at least 21 years old, and experiencing their first diagnosis of stage 0–II breast cancer in the past 3 months. Access to email and Internet on a desktop or laptop computer was required since CaringGuidance™ was not mobile-capable at that time. Clinics were encouraged to distribute flyers to women as early as possible post-diagnosis.

### Procedure

After screening by phone, eligible subjects provided written consent and were randomized to usual care plus CaringGuidance™ (intervention) or to usual care alone (control). Randomization was determined prior to study initiation using a random number generator to create an allocation sequence in blocks of four. Enrollment occurred from August 2013 to August 2015. Four measurement occasions were collected (baseline and months 1, 2, and 3). All monthly data were self-reported and returned by US mail after which subjects received a $25 Amazon gift card [15, 16].

#### Both groups

No restrictions were imposed on usual care. Subjects tracked medical appointments, symptoms, and source of support received to capture usual care during the 12 weeks. All subjects received scripted phone calls from one research assistant (RA) at 28 ± 5 working day intervals to review log entries and assess for adverse events. All calls were digitally recorded, and a 10% sample was reviewed by the PI for script fidelity [15, 16].

#### Intervention group

Subjects were informed that a suggested dose of independent CaringGuidance™ use was 20–30 min, 2–3 times per week (i.e., 40–90 min/week for 12 weeks). This suggested dose was estimated according to the traditional 12 hourly sessions of in-person therapy. A brief one-time orientation to the program’s three introductory pages was provided verbally or by email. Subjects received a pictorial guide on general website use (e.g., increasing volume, font size). Program engagement was encouraged through automatically generated emails. To support intervention receipt, the RA asked scripted questions during the monthly phone call regarding subjects’ perceived ease of program log-in and use. The RA provided a scripted verbal reminder regarding areas of CaringGuidance™ that a subject had not explored [15, 16].

### Measures

Distress, the primary multidimensional [8] outcome, was measured in three ways.

#### Distress Thermometer

The Distress Thermometer (DT) is a single-item, 0–10 scale [26]. The DT is accurate assessing distress when compared with the Hospital Anxiety and Depression Scale and the Brief Symptom Inventory-18 with a score of ≥ 4 of 10 associated with poorer performance status among ambulatory cancer patients, including women with breast cancer [27].

#### Center for Epidemiologic Studies Depressive Scale

The 20-item Center for Epidemiologic Studies Depressive Scale (CES-D) [28] was used to measure depressive symptoms. Higher scores indicate more severe symptoms. Scores ≥ 16 are clinically significant. Internal consistency is alpha = .90 in patient and alpha = .80 in community populations [28]. In the current study, alpha = .86.

#### Impact of Event Scale

Intrusive and avoidant thoughts anchored to the breast cancer diagnosis were measured with the 15-item, 4-point Impact of Event Scale (IES) [29]. A score ≥ 9 indicates an impactful event. Scores ≥ 26 represent strong impact demonstrated by intrusive/avoidant thinking. Cronbach’s alpha for the entire scale equals .86 [29] and in this study, alpha = .87.

#### Demographics and exploratory psychosocial variables

Self-reported demographic variables were collected at baseline including subject’s history of computer use (8-item yes/no), prior breast cancer diagnosis of family/friend (yes/no), health literacy (a single-item “When you go to the doctor’s office, how confident are you filling out medical forms by yourself” (“extremely” to “not at all”) [30]), and a study-derived single-item (yes/no) question on stressful events in past year. At baseline, and again monthly, study-derived questions were used to measure history of mental healthcare (3-item yes/no), with the remainder single-item responses on perceived support in the past week (1 “not at all”–10 “greatly”), level of personally modifiable causal attribution for cancer (0 “not at all” to 5 “extreme”), sense of control over cancer and treatment (0 “not at all” to 5 “extreme”), and self-perception of coping (1 “not well at all”–10 “extremely well”).

Dispositional optimism was measured at baseline because of its well-established association with psychological adjustment [31]. The Life Orientation Test-Revised (LOT-R) [32] was used, in which higher scores on this 10-item scale indicate greater optimism. The LOT-R has alpha = .78 [32], and alpha = .81 in this study.

Coping was measured at baseline and monthly using the Brief COPE [33], a measure of 14 coping responses rated on a 1 (“I haven’t been doing this at all”) to 4 (“I’ve been doing this a lot”) scale. The two-item Active Coping subscale (alpha = .68) [33] was examined for this study in which alpha = .67.

#### Intervention usage

Minutes of use, number of sessions, mean log-in duration, and the type of program material accessed were captured by the CaringGuidance™ data analytics system [16].

### Sample size

Power analysis indicated an estimated sample size of 54 subjects (27/group) for repeated measures ANOVA with four time points, small to medium effect size of .35, average correlation coefficient of .5, and alpha .05 [34]. Effect size was estimated based on prior publications of unguided, web-based CBT interventions for cancer-related distress [25, 35]. Projected attrition was 23% based on our prior work with newly diagnosed women undergoing cancer treatment during psychosocial studies [17, 18]. Additionally, we planned to compare baseline mood differences among women completing baseline measures before versus after primary surgical treatment to inform our future work and an interim analysis was planned to prepare a grant submission. Thus, the target enrollment was set at 100 subjects.

Descriptive statistics were calculated on all study variables. Spearman’s correlations were calculated between demographic and study variables. Due to non-normality in depressive symptoms and impact of events, these variables were transformed using a square root transformation prior to analysis. The primary analyses were performed using multilevel modeling (MLM) [36, 37] (i.e., hierarchical linear modeling, or mixed effects models). Parameter estimates were obtained using restricted maximum likelihood estimation and Kenward-Rogers degrees of freedom for tests of significance [38]. MLM utilizes all available data through the use of maximum likelihood estimation (i.e., no listwise deletion), so all subjects with at least one measurement occasion are used in the analysis. Models included random intercepts and slopes across subjects, as well as an unstructured covariance matrix to estimate the covariance between intercepts and slopes. Direct tests of intervention effects were assessed by time by group interaction. Separate models were performed for each outcome variable. The MIXED procedure in SAS version 9.4 was used for these analyses.

For exploratory tests of moderation, baseline levels of demographic variables of interest based on evidence pertaining to breast cancer-related distress [39] were included to test if the intervention was more or less effective for certain women. The moderators tested were age, income, prior mental health diagnosis, stressful life event in past year, surgical status at baseline (pre/post), breast cancer stage, perceived support, causal attribution, optimism, coping (active and perceived), and baseline distress, depressive symptoms, and impact of cancer event.

## Results

**Fig. 1:**
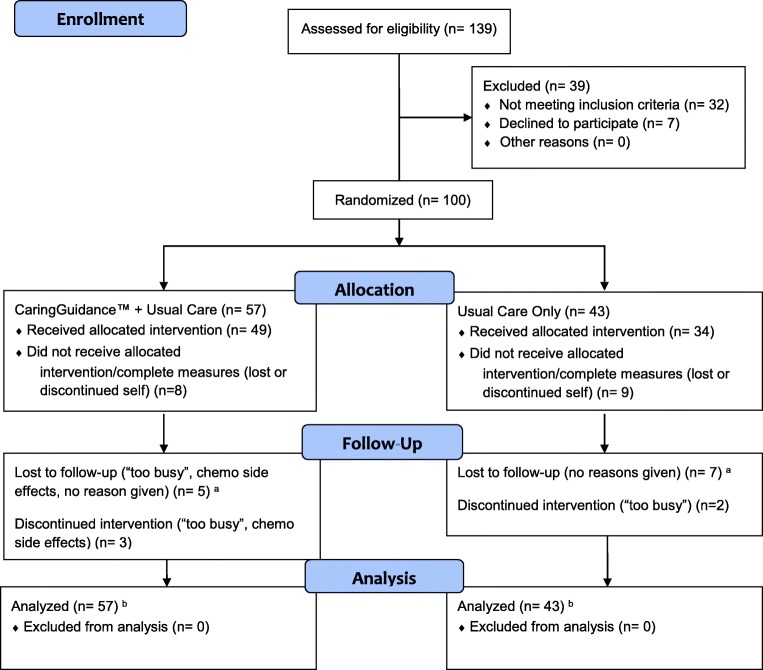
CONSORT flow diagram. A superscript letter “a” denotes that subjects did not complete month 1, month 2, and/or month 3 study measures, and did not withdraw/discontinue. A superscript letter “b” denotes that all subjects allocated to a study condition were included in the analysis

Of 139 women screened, 100 were enrolled and randomly assigned to condition (43 control; 57 intervention). Nine control and eight intervention subjects withdrew or were lost to follow-up resulting in 17% attrition (Fig. 1). Attrition did not bias treatment effects as there were no significant differences between groups on dropout rate, number of time points completed, or the last time point completed [15].

Enrolled subjects resided in 5 states within the Eastern and Midwestern United States [16]. The last subject completed participation in November 2015 (Fig. 1).

### Baseline characteristics

Table 1 provides descriptive demographic, treatment timing, and intervention usage data.

**Table 1:** Baseline characteristics, treatment, and CaringGuidance™ Usage (*N* = 100) [15, 16]

	Intervention (*n* = 57)	Control (*n* = 43)	Total (*n* = 100)	*p* value^a^
Age, years (mean; SD)	55.1 (9.4)	53.2 (10.5)	54.2 (9.9)	.35
Race				
White	53 (93%)	42 (97.7%)	95	.10
African American	3 (5.3%)	0	3	
American Indian/Alaskan Native	1 (1.8%)	0	1	
Asian	0	1 (2.3%)	1	
Ethnicity				
Hispanic	1	0	1	
Marital status				
Married	33 (57.9%)	31 (72.1%)	64	.14
Divorced	12 (21.1%)	7 (16.3%)	19	
Single	6 (10.5%)	3 (7%)	9	
Widowed	4 (7%)	2 (4.7%)	6	
Partnered	2 (3.5%)	0	2	
Highest level of education				
Completed college or graduate school	39 (70.9%)	30 (71.5%)	69	.96
Some college	11 (20%)	9 (21.4%)	20	
Technical training	2 (3.6%)	1 (2.4%)	3	
High School	3 (5.5%)	2 (4.8%)	5	
Missing	2 (3.6%)	1 (2.4%)	3	
Employed				
Full time	36 (63.2%)	22 (51.2%)	58	.21
Part time	10 (17.5%)	6 (14%)	16	
Not employed	11 (19.3%)	15 (34.9%)	26	
Income				
0–$24,999	4 (8%)	2 (4.8%)	6	.04*
$25,000–$49,999	9 (18%)	12 (28.6%)	21	
$50,000–$74,999	9 (18%)	15 (35.7%)	24	
$75,000–$99,999	7 (14%)	6 (14.3%)	13	
> $100,000	21 (42%)	7 (16.7%)	28	
Missing	7 (14%)	1 (2.4%)	8	
Stage at baseline				
0	14 (24.6%)	10 (23.3%)	24	.93
I	25 (43.9%)	19 (44.2%)	44	
II	16 (28.1%)	14 (32.6%)	30	
“early stage”	2 (3.5%)	0	2	
Time since diagnosis				
< 4 weeks	22 (38.6%)	17 (39.5%)	39	.89
1–2 months	28 (49.1%)	22 (51.2%)	50	
2–3 months	7 (12.3%)	4 (9.3%)	11	
Surgical status at completion of baseline measures				
Not completed surgical treatment	33 (57.9%)	33 (76.7%)	66	.049*
Primary surgery complete	24 (42.1%)	10 (23.3%)	34	
No prior mental health diagnosis (*n* = 99)	44 (77.2%)	31 (73.8%)	75	.70
Health literate				
Extremely/quite a bit	54 (94.7%)	43 (100%)	97	.31
Prior Internet use	57 (100%)	43 (100%)	100	NA
Personal attribution for cancer (extremely to somewhat)	16 (28.1%)	12 (28.6%)	28	.95
Stressful life event in prior year	26 (45.6%)	21 (50%)	47	.67
Family member or close friend with history of breast cancer	42 (73.7%)	32 (74.4%)	74	.93
Perceived control over cancer and treatment (extremely to quite a bit)	23 (41.1%)	12 (28.6%)	35	.26
Perceived coping success [1 = not well at all; 10 = extremely well] (mean; SD)	7.2 (1.6)	6.5 (2.0)	6.9 (1.8)	.16
Distress Thermometer (mean; SD)	4.7 (2.1)	4.8 (2.6)	4.76 (2.33)	.81
CES-D (mean; SD)	13.1 (7.6)	14.4 (9.9)	13.67 (8.67)	.50
Impact of Events Scale (mean; SD)	24.2 (13.4)	27.0 (15.7)	25.37 (14.37)	.36
Clinically significant score at baseline				
Distress Thermometer (≥ 4) ^b^	40 (70.2%)	26 (60.5%)	66	.40
CES-D (≥ 16)	20 (35.1%)	16 (37.2%)	36	.76
Impact of Events (≥ 26)	22 (38.6%)	22 (51.2%)	44	.16
CaringGuidance™ usage (*n* = 54) ^c^		na		na
Time, hours (mean; SD)	4.98 (3.61)			
0 sessions	1 (1.9%)			
1–5 sessions	10 (18.5%)			
6–10 sessions	11 (20.4%)			
11–15 sessions	13 (24.1%)			
16–20 sessions	10 (18.5%)			
21–26 sessions	9 (16.7%)			
Breast surgical procedures between				
Baseline to month 1	21 (38.2%)	18 (46.2%)	39	.440
Month 1 to month 2	6 (12%)	4 (10.8%)	10	.863
Month 2 to month 3	3 (6.1%)	7 (20%)	10	.053
Chemotherapy received during study				
Baseline to month 1	11 (20%)	9 (23.1%)	20	.719
Month 1 to month 2	9 (18%)	10 (27%)	19	.314
Month 2 to month 3	13 (26.5%)	14 (40%)	27	.193
Radiation therapy received during study				
Baseline to month 1	10 (18.2%)	6 (15.4%)	16	.722
Month 1 to month 2	17 (34%)	5 (13.5%)	22	.030*
Month 2 to month 3	16 (32.7%)	8 (22.9%)	24	.327
Received clinical support services^d^ Days (mean; SD)				
Baseline to month 1	3.64 (3.15)	3.38 (3.67)	3.53 (3.36)	.561
Month 1 to month 2	2.32 (2.45)	2.76 (2.52)	2.51 (2.48)	.307
Month 2 to month 3	2.10 (3.83)	3.23 (3.45)	2.57 (3.70)	.023*

*p ≤ .05

*CES-D*, Center for Epidemiologic Studies Depression Scale

^a^*p* values reflect the significance of group differences of *χ*^2^ tests for categorical variables and *t* tests for continuous variables

^b^Clinically significant cutoffs

^c^Complete CaringGuidance™ usage data is available on 54 of 57 women due to one woman not receiving a password and two dropouts in M1 prior to logging in. A session is defined as a continuous period of program user activity

^d^Clinical supportive services were defined and documented by participants in daily logs and included emotional, informational, and practical support from healthcare professionals which could be physicians, social work, psychology, etc.

The intervention and control groups did not differ on baseline demographic characteristics with the exception that income was slightly higher in the intervention group (*p* = .042) (Table 1). Income was not correlated with other baseline variables (Table 2). The intervention and control groups did not differ on cancer stage (*p* = .93) nor time since diagnosis (*p* = .89), with 89% of subjects being within two or fewer months of diagnosis at baseline (Table 1). A greater proportion of the control group completed baseline measures prior to receiving breast cancer surgery (76.7%, *n* = 33) than the intervention group (57.9%, *n* = 33) (*p* = .049) (Table 1); however, this did not bias treatment effects because the groups did not differ on baseline distress (i.e., DT, CES-D, and IES) and equal proportions of subjects in each condition also demonstrated clinically significant baseline distress (i.e., DT ≥ 4, CES-D ≥ 16, or IES ≥ 26) (Table 1).

**Table 2:** Baseline Spearman correlation (*n* = 100)

Variable	1	2	3	4	5	6	7	8	9	10	11	12	13	14	15	16
1. Age (years)	–															
2. Income	− 117	–														
3. Health literacy	.014	.180	–													
4. Prior mental health diag.	− .159	− .051	− .062	–												
5. Stressful event prior year	.005	.039	.034	− .019	–											
6. Family/friends prior breast ca	.096	.004	− .078	− .198^a^	.200^a^	–										
7. Personal causal cancer attribution	.079	− .038	.026	.164	.021	− .226^a^	–									
8. Control over cancer and treatment	.133	.126	.223^a^	− .190	.029	.068	− .061	–								
9. Pre-/post-op	.149	.075	.201^a^	− .012	− .049	− .152	.117	.030	–							
10. Felt support in past week	.061	.026	.100	− .088	.057	.129	.010	.158	− .005	–						
11. Perceived coping	.163	.187	.058	− .049	.079	− .071	.029	.408^b^	.117	.404^b^	–					
12. Distress (DT)	− .160	.027	− .082	.124	.161	.114	.047	− .335^b^	− .229^a^	− .236^a^	− .511^b^	–				
13. Depressive symptoms (CES-D)	− .225^a^	− .051	− .033	.258^a^	.071	.125	.155	− .305 ^b^	− .129	− .336^b^	− .590^b^	.625^b^	–			
14. Impact of Event (IES)	− .084	− .076	− .117	.067	.069	.115	.029	− .208^a^	− .180	− .182	− .421^b^	.354^b^	.603^b^	–		
15. Optimism (LOT-R)	.382^b^	.008	.144	− .148	.055	.092	− .109	.364^b^	.028	.341^b^	.303^b^	− .252^b^	− .501^b^	− .334^b^	–	
16. Active coping (Brief COPE)	− .053	.064	.122	.029	.061	.136	.015	.156	.015	.299^b^	.048	.013	− .052	.067	.142	–

*DT*, Distress Thermometer; *CES-D*, Center for Epidemiologic Studies Depression Scale; *IES*, Impact of Event Scale; *LOT-R*, Life Orientation Test-Revised

Pre-op = 0; post-op = 1

^a^*p* ≤ .05

^b^*p* < .01

Table 2 presents the baseline Spearman correlations for demographic and psychosocial variables.

### Usual care (both groups)

Groups did not differ during the study with respect to the months when breast cancer surgery or chemotherapy was received. However, more intervention subjects received radiation during month 2 than control subjects (*p* = .03).

Both groups reported accessing clinical support services from healthcare providers (e.g., physicians, social work, psychology) equally in all study months except month 3 when intervention subjects reported accessing clinical support services fewer days on average than control subjects (*p* = .023) (Table 1).

Groups did not differ (*p* > .05) on duration of the monthly RA phone call thus minimizing potential bias from research staff interactions with subjects [16].

### Intervention use

The intervention group spent 0 (*n* = 1) to 1265 min (*n* = 1) using CaringGuidance™ (*M* = 4.98 h; SD = 3.61). Subjects accessed CaringGuidance™ between 0 and 26 separate sessions per subject (*M* = 15.33 sessions; SD = 9.96) (Table 1). Mean session duration per subject ranged from 0 (*n* = 1) to 72.11 min. All modules, videos, and exercises were accessed by intervention subjects (additional detail previously published [15, 16]).

### Intervention effects

**Fig. 2:**
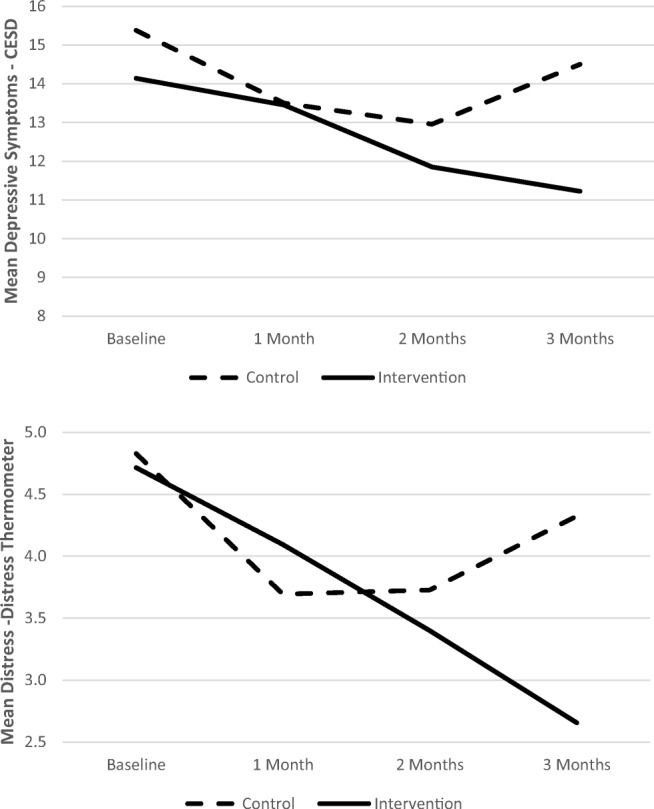
Depressive symptoms and distress for intervention and control groups over 3 months. CES-D Center for Epidemiologic Studies Depression Scale

No significant overall time by group interactions were observed. Post hoc analysis showed significant differences in slopes between groups between study months 2 and 3 on depressive symptoms and distress (measured by the DT). In other words, from baseline to study month 2, both groups experienced a decline in distress, depressive symptoms, and intrusive/avoidant thoughts. However, from study month 2 to month 3, the intervention group continued to decline, whereas the control group experienced an increase on all three measures. The slope difference between groups during the month 2 to month 3 interval was significant for distress measured by DT (*F*(1,70) = 4.91, *p* = .03, *η*^2^ = .065), and depressive symptoms (CES-D) (*F*(1, 76) = 4.25, *p* = .043, *η*^2^ = .053), but not intrusive/avoidant thoughts (IES) (*F*(1,64) = 1.81, *p* = .18, *η*^2^ = .03) (Fig. 2).

### Moderators of intervention effects

**Fig. 3:**
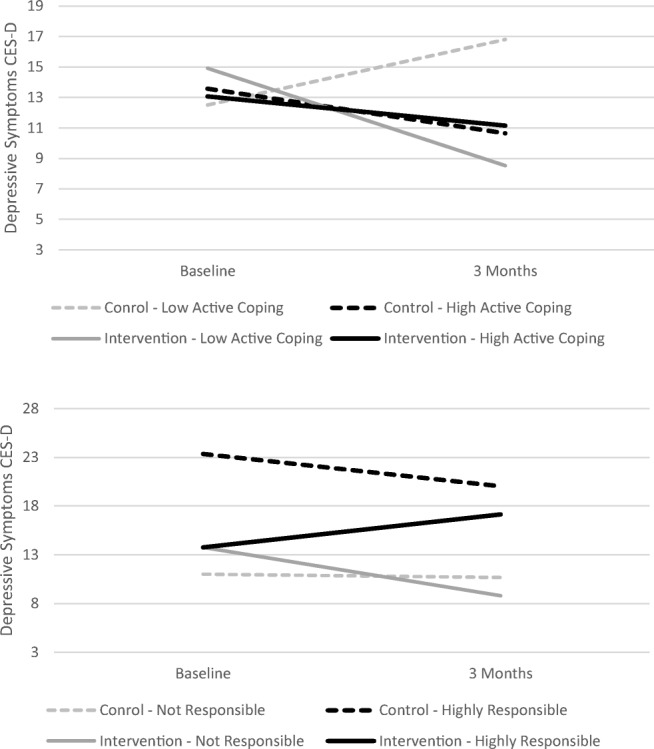
Active coping and personal causal attribution (i.e., responsibility) for diagnosis as moderators of CaringGuidance™ effect on depressive symptoms. CES-D Center for Epidemiologic Studies Depression Scale

**Fig. 4:**
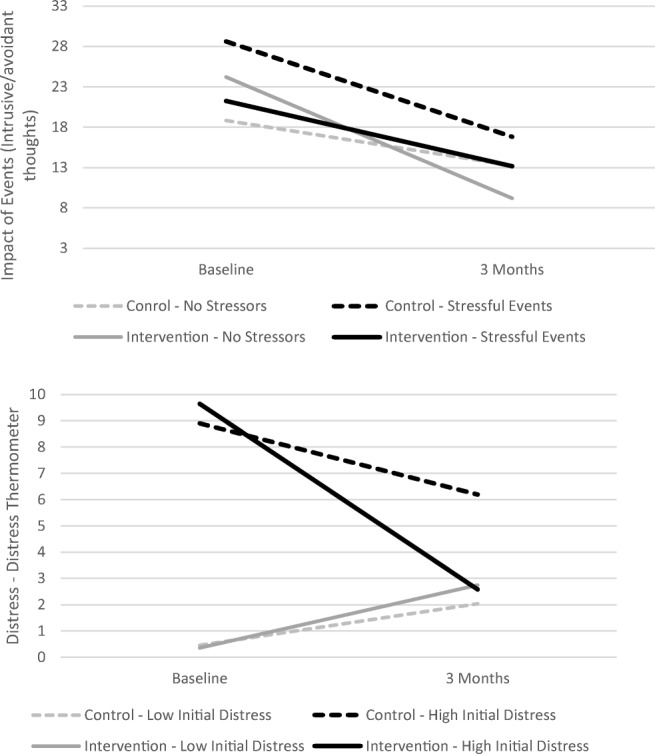
Stressful event in prior year and baseline distress (Distress Thermometer) as moderators of CaringGuidance™ effect on intrusive/avoidant thinking and distress

Exploratory analysis identified four variables that appeared to moderate the effects of CaringGuidance™. Figures 3 and 4 present model-predicted scores based on group and varying levels of the moderators.

The effect of CaringGuidance™ on distress (measured by the DT) varied depending on initial baseline DT score [*t*(77) = − 2.18, *p* = .032]. No difference was observed for subjects low on initial DT distress scores. For subjects with higher DT scores at baseline, however, distress reduction was greater for the intervention than the control group (Fig. 4).

Active coping significantly moderated the effect of the intervention on depressive symptoms [*t*(84.2) = 2.12, *p* = .037] such that while there was no effect of the intervention on depressive symptoms for subjects high in active coping at baseline, for subjects low in active coping, greater reduction in depressive symptoms occurred over time (Fig. 3).

The intervention effect on depressive symptoms was also significantly moderated by personal causal attribution beliefs [*t*(80.8) = 2.02, *p* = .047]. For subjects taking little to no responsibility for their breast cancer, the intervention had a reducing effect on depressive symptoms. But for subjects who felt “somewhat” to “extremely” responsible for their cancer diagnosis, depressive symptoms increased among the intervention group (Fig. 3).

The effect of the intervention on intrusive/avoidant thinking was significantly moderated by whether subjects experienced a stressful event in the prior year [*t*(78.6) = 2.41, *p* = .018]. For subjects who reported a prior stressor, there was no difference in change in the amount of intrusive/avoidant thinking as a result of the intervention. But, for those with no major stressor in the prior year, the intervention group demonstrated reduced intrusive/avoidant thoughts more so than the control group (Fig. 4).

No other baseline variables explored were identified as potential moderators of the intervention.

## Discussion

In this first randomized trial to evaluate the preliminary efficacy of CaringGuidance™, 100 women residing in five US states, diagnosed with early-stage breast cancer in the prior 3 months, were randomized to 12 weeks of unguided use of CaringGuidance™ plus usual care (intervention) or usual care alone (control). As anticipated, both the intervention and control groups experienced reductions in distress, including depressive symptoms and intrusive/avoidant thoughts over the study period. However, preliminary support for the efficacy of CaringGuidance™ was provided as a product of the control group’s increase in distress and depressive symptoms and the intervention group’s decrease in distress and depressive symptoms between months 2 and 3. This outcome could not be explained by differences at baseline, timing of the receipt of cancer treatment during the trial, or clinical supportive services utilized since the control group, for whom distress and depressive symptoms increased, received fewer treatments and accessed more supportive services compared with the intervention group during the period in which the group differences were observed. These results extend what we previously reported on the intervention group’s use of and satisfaction with CaringGuidance™ [16].

These findings also contribute to the limited research on the efficacy of unguided web-based interventions specifically aimed at breast cancer-related distress. Additionally, this may be the first report of a web-based CBT intervention for women initiated within the first months of diagnosis. This work is of value given that BCS are the largest group of cancer survivors in the USA [1], and unguided interventions which by their nature do not rely on clinical resources are potentially sustainable [21] while also being efficacious in mental healthcare [22, 40].

Baseline psychosocial characteristics of our sample reflect those in published research [39, 41, 42] such that greater optimism, and perceived coping, were negatively associated with distress and depressive symptoms and, as expected, younger age and depressive symptoms were also negatively associated [41]. Contrary to existing evidence [39] however, younger age was not related to greater distress (DT) or intrusive/avoidant thoughts. This finding may be indicative of the study’s small sample and will be explored in future studies of this intervention.

The study results are consistent with the other two existing web-based unguided programs specifically for breast cancer with regard to distress reduction. However, the BREATH trial, recently published from the Netherlands, enrolled women who had completed cancer treatment 2–4 months before enrollment [24], and contrary to BREATH, we saw a potentially greater benefit of the intervention for women who were highly distressed at baseline, whereas the BREATH found the opposite [24]. Given the exploratory and pilot-nature of our study, this outcome should be examined further in a larger sample of women.

Our results are also consistent with the 12-week unguided group intervention for longer-term BCS, SURVIVE, in that neither study identified significant effects on distress as measured by the IES [24]. However, in contrast, CaringGuidance™ demonstrated preliminary efficacy with respect to distress manifesting as depressive symptoms while neither the BREATH [24] nor SURVIVE studies [25] measured depressive symptoms. Given the long-term burden of depression among BCS [7], further study of CaringGuidance™’s potential to reduce depressive symptoms in newly diagnosed women and the role active coping may play in moderating these effects is warranted.

Finally, our findings point to a need to further explore the relationship of CaringGuidance™ and causal attribution among newly diagnosed women. Evidence on the effects of causal attribution beliefs on psychological adjustment to breast cancer is inconsistent [43, 44]. In this study, exploration of potential modifiers of CaringGuidance™ effects identified that women with access to the intervention who held high personally modifiable causal attribution beliefs about their cancer experienced an increase in depressive symptoms as compared with women with low personal causal attributions. The reason for this is unclear and warrants additional study. Women were asked in this study to rate the degree to which they attributed the cancer diagnosis to their actions or inactions, but not the specific attribution or why they held their beliefs. Despite CaringGuidance™ content on the improbability that a single personal action or inaction results in a woman’s breast cancer, it is possible that certain beliefs could not be overcome to affect women’s depressive symptoms.

Several limitations of this study must be considered. First, the sample size was based on estimated effect size from the literature and may have resulted in a lack of power to detect interaction effects. Second, multiple potential intervention modifiers were explored with the aim of informing our future work, and thus these results were viewed with caution given the number of modifiers tested. Furthermore, in order to reduce subject burden, we opted to use single-item, study-derived questions to measure causal attribution and stressful events in the prior year rather than using lengthier existing tools which may have affected results. Nevertheless, these findings inform us of the need to further explore these variables with valid instruments in a future larger study to see whether these exploratory outcomes hold. Third, no lower limit was placed on baseline distress for study inclusion, potentially resulting in a floor effect for subjects without initial clinically significant symptoms. Future studies will require all subjects to have a clinically significant score on the DT, CES-D, or IES at baseline. Furthermore, despite our attempts to recruit African American women, the sample is primarily White and therefore not representative of all US women with breast cancer. Ability to read English was also required because CaringGuidance™ was developed in English and for the most part, subjects were college-educated and computer-experienced. While the sample characteristics may limit generalizability, this sample is typical of samples of women who participate in trials published within breast cancer psychosocial literature. Finally, although the scripted monthly phone calls were made by one RA to all subjects, future results may differ without this contact.

## Conclusion

This study provides initial support for the preliminary efficacy of CaringGuidance™ when compared with usual care alone, as an unguided, web-based, self-management tool for breast cancer-related distress. Particularly promising for future study are exploratory outcomes favoring greater distress reduction for women with program access who either infrequently used active coping strategies or experienced high levels of distress after diagnosis. Together with the feasibility and satisfaction outcomes reported earlier [16], the current findings highlight the promise of CaringGuidance™ to contribute to filling a current gap in distress management for women with newly diagnosed, early-stage breast cancer and warrant further study of this intervention.

## References

[1] DeSantis CE, Ma J, Sauer AG, Newman LA, Jemal A (2017). Breast cancer statistics, 2017, racial disparity in mortality by state. CA Cancer J Clin.

[2] Zabora J, Brintzenhofeszoc K, Curbow B, Hooker C, Piantadosi S (2001). The prevalence of psychological distress by cancer site. Psychooncology.

[3] Acquati C, Kayser K (2017). Predictors of psychological distress among cancer patients receiving care at a safety-net institution: the role of younger age and psychosocial problems. Support Care Cancer.

[4] Linden W, Vodermaier A, MacKenzie R, Greig D (2012). Anxiety and depression after cancer diagnosis: prevalence rates by cancer type, gender, and age. J Affect Disord.

[5] Burgess C, Cornelius V, Love S, Graham J, Richards M, Ramirez A (2005). Depression and anxiety in women with early breast cancer: five year observational cohort study. BMJ.

[6] Tojal C, Costa R (2015). Depressive symptoms and mental adjustment in women with breast cancer. Psychooncology.

[7] Maass SWMC, Roorda C, Berendesen AJ, Berhaak PFM, deBock GH (2015). The prevalence of long-term symptoms of depression and anxiety after breast cancer treatment: a systematic review. Maturitas.

[8] National Comprehensive Cancer Network. NCCN Guidelines for Distress Management Version 3.2019. www.NCCN.org. Accessed 18 June 2019

[9] Stagl JM, Bouchard LC, Lechner SC, Blomberg BB, Gudenkauf LM, Jutagir DR, Gluck S, Derhagopian RP, Carver CS, Antoni MH (2015). Long-term psychological benefits of cognitive-behavioral stress management for women with breast cancer: 11-year follow-up of a randomized controlled trial. Cancer.

[10] Brandao T, Schulz MS, Matos PM (2017). Psychological adjustment after breast cancer: a systematic review of longitudinal studies. Psychooncology.

[11] Zebrack B, Kayser K, Padgett L, Sundstrom L, Jobin C, Nelson K, Fineberg I (2016). Institutional capacity to provide psychosocial oncology support services: a report from the association of oncology social work. Cancer.

[12] Rankin NM, Butow PN, Thein T, Robinson T, Shaw JM, Price MA, Clover K, Shaw T, Grimison P (2015). Everybody wants it done but nobody wants to do it: an exploration of the barrier and enablers of critical components towards creating a clinical pathway for anxiety and depression in cancer. BMC Health Serv Res.

[13] Mitchel AJ (2013). Screening for cancer-related distress: when is implementation successful and when is it unsuccessful?. Acta Oncol.

[14] Lally RM, McNees P, Meneses K (2015). Application of a novel transdisciplinary communication technique to develop an internet-based psychoeducational program. Appl Nurs Res.

[15] Lally Robin M., Kupzyk Kevin, Mills Adam, Gallo Steven, Meneses Karen (2019). Effects of social constraints and web-based psychoeducation on cancer-related psychological adjustment early-after breast cancer diagnosis. Journal of Psychosocial Oncology.

[16] Lally R, Bellavia G, Gallo S, Kupzyk K, Helgeson V, Brooks C, Erwin D, Brown J (2019). Feasibility and acceptance of the CaringGuidance™ web-based, distress management, psychoeducational program initiated within 12-weeks of cancer diagnosis. Psychooncology.

[17] Lally RM (2010). Acclimating to breast cancer: a process of maintaining self-integrity in the pretreatment period. Cancer Nurs.

[18] Lally RM, Hydeman J, Schwert K, Henderson H, Edge S (2012). Exploring the first days of adjustment to cancer: a modification of acclimating to breast cancer theory. Cancer Nurs.

[19] Heber E, Ebert DD, Lehr D, Cuijpers P, Berking M, Nobis S, Riper H (2017). The benefit of web-and computer-based interventions for stress: a systematic review and meta-analysis. J Med Internet Res.

[20] Ye M, Du K, Ahou J, Zhou Q, Shou M, Hu B, Jiang P (2018). A meta-analysis of the efficacy of cognitive behavior therapy on quality of life and psychological health of breast cancer survivors and patients. Psychooncology.

[21] Baumeister H, Reichler L, Munzinger M, Lin J (2014). The impact of guidance on internet-based mental health interventions — a systematic review. Internet Interv.

[22] Morgan C, Mason E, Newby JM, Mahoney AEJ, Hobbs MJ, McAloon J, Andrews G (2017). The effectiveness of unguided internet cognitive behavioural therapy for mixed anxiety and depression. Internet Interv.

[23] Beatty L, Kemp E, Coll JR, Turner J, Butow P, Milne D, Yates P (2019). Finding my way: results of a multicentre RCT evaluating a web-based self-guided psychosocial intervention for newly diagnosed cancer survivors. Support Care Cancer.

[24] van den Berg SW, Gielissen MFM, Custers JAE, van der Graaf WTA, Ottevanger PB, Prins JB (2015). BREATH: web-based self-management for psychological adjustment after primary breast cancer: results of a multicenter randomized controlled trial. JCO.

[25] Owen JE, Klapow JC, Roth DL (2005). Randomized pilot of a self-guided internet coping group for women with early-stage breast cancer. Ann Behav Med.

[26] Roth AJ, Kornblith AB, Batel-Copel L, Peabody E, Scher HI, Holland JC (1998). Rapid screening for psychological distress in men with prostate carcinoma: a pilot study. Cancer.

[27] Jacobsen PB, Donovan KA, Trask PC, Fleishman SB, Zabora J, Baker F, Holland JC (2005). Screening for psychologic distress in ambulatory cancer patients. Cancer.

[28] Radloff LS (1977). The CES-D scale: a self-report depression scale for research in the general population. Appl Psychol Meas.

[29] Horowitz M, Wilner N, Alvarez W (1979). Impact of events scale: a measure of subjective stress. Psychosom Med.

[30] Wallace LS, Rogers ES, Roskos SE, Holiday DB, Weiss BD (2006). Brief report: screening items to identify patients with limited health literacy skills. J Gen Intern Med.

[31] Matthews EE, Cook PF (2009). Relationships among optimism, well-being, self-transcendence, coping and social support in women during treatment for breast cancer. Psychooncology.

[32] Scheier MF, Carver CS, Bridges MW (1994). Distinguishing optimism from neuroticism (and trait anxiety, self-mastery, and self-esteem): a re-evaluation of the Life Orientation Test. J Pers Soc Psychol.

[33] Carver CS (1997). You want to measure coping but your protocol’s too long: consider the Brief COPE. Int J Behav Med.

[34] Stevens JP (2002). Applied multivariate statistics for the social sciences.

[35] Beatty L, Koczwara B, Wade T (2011). Cancer coping online: a pilot trial of a self-guided CBT internet intervention for cancer-related distress. EJ Appl Psychol.

[36] Raudenbush SW, Bryk AS (2002). Hierarchical linear models: applications and data analysis methods.

[37] Snijders TAB, Bosker RJ (1999). Multilevel analysis: an introduction to basic and advanced multilevel modelling.

[38] Kowalchuk RK, Keselman HJ, Algina J, Wolfinger RD (2004). The analysis of repeated measurements with mixed-model adjusted F tests. Educ Psychol Meas.

[39] Syrowatka A, Motulsky A, Kurteva S, Hanley JA, Dixon WG, Meguerditchian AN, Tamblyn R (2017). Predictors of distress in female breast cancer survivors: a systematic review. Breast Cancer Res Treat.

[40] Newman MG, Szkodny LE, Sj L, Przeworski A (2011). A review of technology-assisted self-help and minimal contact therapies for anxiety and depression: is human contact necessary for therapeutic efficacy?. Clin Psychol Rev.

[41] Friedman LC, Kalidas M, Elledge R, Chang J, Romero C, Husain I, Fulay MF, Liscum KR (2006). Optimism, social support and psychosocial functioning among women with breast cancer. Psychooncology.

[42] Yu Y, Sherman KA (2015). Communication avoidance, coping and psychological distress of women with breast cancer. J Behav Med.

[43] Friedman LC, Romero C, Elledge R, Chang J, Kalidas M, Dulay MF, Lynch GR, Osborne CK (2007). Attribution of blame, self-forgiving attitude and psychological adjustment in women with breast cancer. J Behav Med.

[44] Ferrucci LM, Cartmel B, Turkman YE, Murphy ME, Smith T, Stein KD, McCorkle R (2011). Causal attribution among cancer survivors of the ten most common cancers. J Psychosoc Oncol.

